# Contribution of the galanin system to inflammation

**DOI:** 10.1186/2193-1801-4-S1-L57

**Published:** 2015-06-12

**Authors:** Barbara Kofler, Susanne Brunner, Andreas Koller, Silke Wiesmayr, Felix Locker, Roland Lang, Balint Botz, Àgnes Kemény, Zsuzsanna Helyes

**Affiliations:** Laura Bassi Centre of Expertise THERAPEP, Research Program for Receptor Biochemistry and Tumor Metabolism, Department of Pediatrics, Paracelsus Medical University, Salzburg, Austria; Department of Pediatrics, Paracelsus Medical University, Salzburg, Austria; Department of Pharmacology and Pharmacotherapy, János Szentágothai Research Center and MTA NAP B Pain Research Group University of Pécs, Pécs, Hungary

**Keywords:** galanin, inflammation, immune cells

Neurogenic inflammatory components mediated by peptidergic sensory nerves have a crucial impact on the symptoms of inflammatory diseases. Galanin is a regulatory sensory neuropeptide, which was shown to attenuate neurogenic inflammation, but our current understanding about its endogenous targets, and physiologic importance is incomplete. Among the endogenous receptors of galanin (GAL1, GAL2, GAL3) we found GAL3 to be the most abundantly expressed on the vasculature and GAL2 on different types of immune cells including polymorphonuclear neutrophils and natural killer cells. Therefore, we evaluated if galanin exerts direct or indirect effects on these immune cells. Our data revealed that galanin can be regarded as an immunomodulatory peptide as it can sensitize polymorphonuclear neutrophils and natural killer cells towards proinflammatory cytokines. Since there are only scarce in vivo data concerning the role of GAL3 in inflammatory disease conditions, we analysed its involvement in the K/BxN serum transfer model of autoimmune arthritis and the oxazolone-model of allergic contact dermatitis, employing GAL3 gene-deficient mice. After arthritis induction, GAL3-knockout mice demonstrated increased clinical disease severity and earlier hindlimb edema than wildtype mice. Vascular hyperpermeability was also elevated compared to wildytpes, but neutrophil myeloperoxidase activity and arthritic hyperalgesia were not significantly different. In contrast, disease severity, vascular, and immune components were not affected in allergic contact dermatitis in GAL3 knockouts in comparison with wildtypes. Our findings suggest GAL3 activation as a substantial anti-inflammatory pathway in neutrophil-dominated autoimmune arthritis, modulating the early neurogenic vascular hyperpermeability and consequent edema formation. However, the involvement of GAL3 activation in the T-cell dependent allergic contact dermatitis remains unsupported.

